# Application of group smoothly clipped absolute deviation method in identifying correlates of psychiatric distress among college students

**DOI:** 10.1186/s12888-020-02591-3

**Published:** 2020-05-04

**Authors:** Mahya Arayeshgari, Leili Tapak, Ghodratollah Roshanaei, Jalal Poorolajal, Ali Ghaleiha

**Affiliations:** 1 Department of Biostatistics, School of Public Health, Hamadan University of Medical Sciences, Hamadan, Iran; 2 Modeling of Noncommunicable diseases Research Center, Hamadan University of Medical Sciences, Hamadan, Iran; 3 Department of Epidemiology, School of Public Health, Hamadan University of Medical Sciences, Hamadan, Iran; 4 Research Center for Health Sciences, Hamadan University of Medical Sciences, Hamadan, Iran; 5 Department of Psychiatry, School of Medicine, Hamadan University of Medical Sciences, Hamadan, Iran; 6 Research Center for Behavioral Disorders and Substance Abuse, Hamadan University of Medical Sciences, Hamadan, Iran

**Keywords:** Smoothly clipped absolute deviation, LASSO, Psychiatric distress, Mental disorders, Substance-related disorders, Suicide, Smoking

## Abstract

**Background:**

College students are at an increased risk of psychiatric distress. So, identifying its important correlates using more reliable statistical models, instead of inefficient traditional variable selection methods like stepwise regression, is of great importance. The objective of this study was to investigate correlates of psychiatric distress among college students in Iran; using group smoothly clipped absolute deviation method (SCAD).

**Methods:**

A number of 1259 voluntary college students participated in this cross-sectional study (Jan-May 2016) at Hamadan University of Medical Sciences, Iran. The data were collected using a self-administered questionnaire consisting of demographic information, a behavioral risk factors checklist and the GHQ-28 questionnaire (with a cut-off of 23 to measure psychiatric distress, recommended by the Iranian version of the questionnaire). Penalized logistic regression with a group-SCAD regularization method was used to analyze the data (α = 0.05).

**Results:**

The majority of students were aged 18–25 (87.61%), and 60.76% of them were female. About 41% of students had psychiatric distress. Significant correlates of psychiatric distress among college students selected by group-SCAD included the average grade, educational level, being optimistic about future, having a boy/girlfriend, having an emotional breakup, the average daily number of cigarettes, substance abusing during previous month and having suicidal thoughts ever (*P* < 0.05).

**Conclusions:**

Penalized logistic regression methods such as group-SCAD and group-Adaptive-LASSO should be considered as plausible alternatives to stepwise regression for identifying correlates of a binary response. Several behavioral variables were associated with psychological distress which highlights the necessity of designing multiple factors and behavioral changes in interventional programs.

## Background

Nearly one-third of college students have been estimated to be involved in mental disorders [1]. College period can be considered as an exciting time for many students; nevertheless, it can be a critical developmental period during one’s lifespan due to the susceptibility to the occurrence of mental disorders. Mental disorders may profoundly affect several aspects of the future life of students, including role impairment investigated by Alonso et al. [2], academic outcome like college attrition investigated by Auerbach et al. [3] and grades investigated by Bruffaerts et al. [4] as well as the development of suicidal thoughts and behaviors investigated by Mortier et al. and Mortier et al. [5, 6]. Mental disorders also expose adolescents and young adults at a higher risk of serious types of disabilities [7] and leaving the disorder untreated can cause severe impairments in college students’ functioning and their subsequent development [8]. “These long-term adverse outcomes may be mediated by mental health problems that exist during the college years, as these years constitute a peak period for the first onset of a broad range of mental disorders” [4].

It has been reported that academic/financial pressures on college students as well as irregular sleep patterns, long hours of study and living away from home for the first time can increase the risk of mental illnesses [9–11]. Mental disorders can have different manifestations, including distorted thoughts, altered perceptions, impaired emotions, abnormal behavior and atypical communication [7, 12]. Some common types of mental health problems among college students include depression, anxiety, substance abuse and eating disorders [8, 10].

Various individual-oriented and socially-oriented factors may contribute to establishing mental disorders. For example, some potential factors include substance abuse, high-risk sexual behaviors and suicidal behaviors/thoughts [7]. While several studies have investigated the factors associated with mental disorders worldwide [13–16], few studies have been conducted about the correlates of such mental illnesses among college students, especially in developing countries including Iran. This highlights the necessity of investigating correlates of mental disorders.

Selecting variables correlated with a binary response, like having/not having a mental disorder, is usually conducted through the stepwise logistic regression procedure [17–20]. However, ad hoc stepwise selection procedures suffer from several shortcomings, where the instability of the selected variables is the most important issue [21], especially when there is a large number of explanatory variables. They also are computationally expensive and most importantly stochastic errors are neglected during the variable selection process of the previous steps [22]. Moreover, stepwise variable selection is prone to “overfitting” the data due to searching in a large space among possible models [23]. So, they may not provide optimal results, making the use of these methods unreliable in practice. Recently, several variable selection methods have been proposed, including regularized techniques where penalties are imposed on the regression coefficients in the likelihood function. Therefore, variable selection and estimation of regression coefficients are done simultaneously. Among different penalties, that have been proposed until now, “smoothly clipped absolute deviation”)SCAD), proposed by Fan and Li [22], has been extensively developed for different regression problems, including logistic regression, which is reported to produce more reliable results and provide unbiased estimates [22, 24].

As the occurrence of psychiatric distress during college life can have severe consequences on different aspects of students’ life, investigating its correlates using more reliable statistical methods is of great importance. Therefore, this study aimed to identify associated correlates of psychiatric distress among college students using penalized logistic regression with the SCAD penalty. We also considered the least absolute shrinkage and operator (LASSO) and typical stepwise logistic regression and compared their performances through a simulation study and a real dataset analysis.

## Methods

### Data source

This cross-sectional study included 1259 college students of Hamadan University of Medical Sciences, from Jan to May 2016. This study has been approved by the Research Council of Hamadan University (IR.UMSHA.REC.1398.075). The data collection tools included: (1) a demographic characteristics/personal information checklist consisting of sex (male/female), age (year), marital status (never married/married/divorced), city (hometown/surrounding towns/towns of other provinces), residence (dormitory/parents’ house), birth order, father’s educational level and mother’s educational level (Diploma, BSc, MSc, Ph.D.); (2) a checklist for educational information consisting of college (study field), the average grade of the previous semester and student’s education level (BSc, MSc, Ph.D.); (3) questions about interest in the discipline and being optimistic about the future; (4) behavioral variables including having a boy/girlfriend, having an emotional breakup, having homosexual intercourse, having heterosexual intercourse, smoking during the previous month, the average daily number of cigarettes, substance abuse ever/previous month/previous year, having suicidal thoughts ever/previous month/previous year, having suicide attempt ever/previous month/previous year and hours of using social networks during a day; and (5) a validated Persian version of the GHQ-28 questionnaire (provides scores ranged from 0 to 84). A cutoff point of 23 was used to determine if a student has/has not psychiatric distress, because the cutoff of 23 was used to discriminate clinical significance, with scores greater than 23 suggestive of psychiatric disturbances, provided for the Iranian version of the questionnaire [25]. All variables were selected based on the literature review and previous studies.

The psychiatric distress was considered as the outcome of interest (binary response variable). Descriptive statistics regarding the characteristics of the college students were provided in Table 1 (for the characteristics of the students with and without psychiatric distress separately see [7], Table 2).

**Table 1:** Demographic/personal characteristics and behavioral variables of students participated in the study (*n* = 1259)

Variable	n (%)	Variable	n (%)
**Sex**		**Optimistic about the future**	
Male	494 (39.24)	Yes	998 (79.27)
Female	765 (60.76)	No	261 (20.73)
**Age group**		**Having a boy/girlfriend**	
18–21	553 (43.92)	Yes	651 (51.71)
22–25	550 (43.69)	No	608 (48.29)
26–29	112 (8.90)	**Having an emotional breakup**	
≥ 30	44 (3.49)	Yes	420 (33.36)
**Marital status**		No	839 (66.64)
Never married	1052 (83.56)	**Having homosexual intercourse**	
Married	164 (13.03)	Yes	100 (7.94)
Divorced	43 (3.41)	No	1159 (92.06)
**City**		**Having heterosexual intercourse**	
Hometown	382 (30.34)	Yes	166 (13.19)
Surrounding towns	396 (31.45)	No	1093 (86.81)
Towns of other provinces	481 (38.21)	**Smoking during the previous month**	
**Residence**		Yes	158 (12.55)
Dormitory	889 (70.61)	No	1101 (87.45)
Parents’ house	370 (29.39)	**Number of cigarettes per day**	
**Birth order**		Non-smoker	1064 (84.51)
1	445 (35.35)	1–9	162 (12.87)
2	397 (31.53)	≥ 10	33 (2.62)
3	228 (18.11)	**Substance abuse ever**	
≥ 4	189 (15.01)	Yes	124 (9.85)
**Father’s educational level**		No	1135 (90.15)
Diploma	592 (47.02)	**Substance abuse previous month**	
BSc	436 (34.63)	Yes	85 (6.75)
MSc	166 (13.19)	No	1174 (93.25)
MD	65 (5.16)	**Substance abuse previous year**	
**Mother’s educational level**		Yes	52 (4.13)
Diploma	805 (63.94)	No	1207 (95.87)
BSc	313 (24.86)	**Having suicidal thoughts ever**	
MSc	108 (8.58)	Yes	204 (16.20)
MD	33 (2.62)	No	1055 (83.80)
**College (study field)**		**Having suicidal thoughts previous month**	
Medicine	366 (29.07)	Yes	94 (7.47)
Dentistry	103 (8.18)	No	1165 (92.53)
Public health	245 (19.46)	**Having suicidal thoughts previous year**	
Paramedical	249 (19.78)	Yes	126 (10.00)
Pharmacology	83 (6.59)	No	1133 (90.00)
Nursing/Midwifery	162 (12.87)	**Having suicide attempt ever**	
Rehabilitation	51 (4.05)	Yes	104 (8.26)
**The average grade of the previous semester**		No	1155 (91.74)
< 14	176 (13.98)	**Having suicide attempt previous month**	
14–15.99	55 (4.37)	Yes	49 (3.90)
16–17.99	359 (28.52)	No	1210 (96.10)
≥ 18	669 (53.13)	**Having suicide attempt previous year**	
**Educational level**		Yes	47 (3.73)
BSc	599 (47.58)	No	1212 (96.27)
MSc	96 (7.63)	**Hours of using social networks per day**	
MD	520 (41.30)	0	157 (12.47)
Ph.D.	44 (3.49)	0.5–5	794 (63.07)
**Interest in the discipline**		≥ 6	308 (24.46)
Yes	1030 (81.81)		
No	229 (18.19)

**Table 2:** Comparison of variable selection methods using diagnostic accuracy over 1000 repetitions for the testing set

Variable selection method	Sensitivity^**a**^	Specificity^**a**^	LR + ^a**p**^	LR-^**bn**^	Total Accuracy^a^
**SCAD**	0.494	0.852	3.659	0.595	0.704
**LASSO**	0.499	0.849	3.660	0.589	0.705
**Stepwise**	0.516	0.151	0.609	3.350	0.302

^a^Greater is better; ^b^Smaller is better; ^p^Positive likelihood ratio; ^n^Negative likelihood ratio

### Data pre-processing and dealing with missing values

Before conducting any analysis, the data were checked for any spelling errors and other irregularities/irrelevancies. So, outliers were removed or corrected, if there were any. In this study, we used boxplots for continuous variables to detect outliers. As there were a number of missing values for some of the variables (missing values were observed in 12 variables, ranged from 0.079 to 0.556%), we used a simple imputation strategy (the mean value was used to impute quantitative variables and the median was used to impute qualitative variables).

### Statistical analysis

The data related to the participants were collected and a penalized logistic regression was utilized to select important correlates of psychiatric distress. We used the group SCAD and the group adaptive LASSO penalties in the logistic regression model to deal with the categorical covariates with more than two categories to select correlates and measure the associations between psychiatric distress and demographic characteristics, personal information and behavioral correlates. Briefly, these models are regression shrinkage and selection approaches that impose different l_1_ penalties on the regression coefficients.

Consider the covariate vector of *X* = (1, X_1_, …, X_*p*_). The usual logistic regression model is defined as follows:

1 $$ P\left({y}_i=1|{x}_i\right)=\pi \left({x}_i^{\prime}\boldsymbol{\beta} \right)=\frac{\exp \left({x}_i^{\prime}\boldsymbol{\beta} \right)}{1+\exp \left({x}_i^{\prime}\boldsymbol{\beta} \right)},\kern0.6em 1\le i\le n, $$

Then, the group SCAD and the group LASSO penalties are attached to the log-likelihood of the logistic regression for Y (binary response variable). In this case, the penalized logistic log-likelihood function becomes as follows:

2 $$ Q\left(\beta; \lambda, \gamma \right)=-{\mathrm{n}}^{-1}\sum \limits_{i=1}^n\left\{{y}_i\log \pi \left({x}_i^{\prime}\boldsymbol{\beta} \right)+\left(1-{y}_i\right)\log \left[1-\pi \left({x}_i^{\prime}\boldsymbol{\beta} \right)\right]\right\}+\sum \limits_{j=1}^p\rho \left(\left\Vert {\beta}_j\right\Vert; \sqrt{d_j}\lambda, \gamma \right) $$

where *ρ*(.) stands for the used penalty, *λ* > 0 is the tuning parameter that plays an important role in selecting variables and *γ* > 2 is the regularization parameter and $\boldsymbol{\beta} =\left({\beta}_0,{\beta}_1^{\prime },\dots, {\beta}_p^{\prime}\right)$ is the vector of regression coefficients. The role of *d*_*j*_ is to provide a proportional amount of regularization according to the size of the j^th^ group.

The SCAD penalty is defined as follows:

3 $$ {\rho}_{SCAD}\left(\beta; \lambda, \gamma \right)=\left\{\begin{array}{l}\lambda \left|\beta \right|,\kern4.079998em if\;\left|\beta \right|\le \lambda, \\ {}\frac{2\gamma \lambda \left|\beta \right|-\left({\beta}^2+{\lambda}^2\right)}{2\left(\gamma -1\right)},\kern0.36em if\;\lambda <\left|\beta \right|\le \gamma \lambda, \\ {}\frac{\lambda^2\left({\gamma}^2-1\right)}{2\left(\gamma -1\right)},\kern2.4em if\;\left|\beta \right|>\gamma \lambda .\end{array}\right. $$

The group version of the SCAD penalty can be found in Wang, Chen, and Li [24] which was proposed for handling categorical variables.

The group LASSO penalty [26] is defined as follows:

4 $$ Q\left(\beta; \lambda \right)=\log L+\sum \limits_{j=1}^p{\rho}_{LASSO}\left(\left\Vert {\beta}_j\right\Vert; \sqrt{d_j}\lambda \right) $$

where *ρ*_*LASSO*_(*β*; *λ*) = *λ*|*β*|.

In the penalized approach, variable selection and parameter estimation are done simultaneously. The used penalized models enjoy the oracle properties. This means that if we know in advance that the true model depends only on a subset of the correlates, these selection methods can identify the right subset model and can provide estimators that satisfy the asymptotic normality assumption [22, 27]. Moreover, in the presence of collinearity problem, these methods have been shown to provide a reduction in the variability of the estimates [28].

To use adaptive group LASSO, first, we used the binary logistic regression model to obtain non-zero coefficients for each variable and computed the adaptive weights as their inverse (w = 1/coefficient). This allows for allocating smaller weights, in the penalty, to the variables with large standardized regression coefficients (as they may be more likely to be correlated).

When using the group SCAD and adaptive group LASSO, there is a non-negative penalty parameter, λ, to determine the magnitude of the penalties of the regression coefficients of the used correlates. When λ is zero, no penalty or shrinkage is imposed on the regression coefficients of the correlates, and the model is just the ordinary logistic regression using all the correlates; when it is large enough, maximum shrinkage is imposed, yielding a model with all regression coefficients equal to zero; when λ takes some values in between, some coefficients will be 0 and some will be nonzero, and the final model is the penalized logistic regression. Correlates with non-zero coefficients are “selected” by the group SCAD and adaptive group LASSO. In this way, the methods select variables that may be associated with psychiatric distress. In this study, to find the optimum value of the tuning parameter, a 10-fold cross-validation strategy was utilized. So, first of all, we divided the total data into two subsets of training and testing sets (a 70 and 30 strategy for the training and testing sets, respectively). The testing set was left out for external validation of the three different methods of the traditional stepwise method, group LASSO, and group SCAD. Then, we split the training data set into 10 subsets randomly and the penalized models were fitted 10 times, each time one out of 10 subsets was left out as the testing set and the other 9 subsets were considered as the training set. Then, the models were implemented using a range of λs which was started from zero (no shrinkage) to a value that puts maximum shrinkage and the λ with the smallest Bayesian Information Criterion (BIC) over the testing sets over 10 times repetitions was chosen. Finally, the method was repeated 1000 times and the estimated coefficients were averaged over all repetitions. To estimate standard errors of the coefficients, a bootstrap strategy was used with 1000 replications. So, 1000 samples (with replacement) were selected from the original data and then the standard errors of the coefficients were computed to calculate the two-sided *P*-values. A significance level of 0.05 was considered for all statistical analyses.

For the sake of comparison, in this study, the stepwise logistic regression model was also used. To compare the penalized methods and stepwise approach, we divided the data set into two sets of training and testing. The models were applied to the training set 1000 times and the prediction accuracy of the models was investigated on the testing set using five criteria, including sensitivity, specificity, positive likelihood ratio (LR+), negative likelihood ratio (LR-) and total accuracy. For more investigation, we also conducted a simulation study. In the simulation study, we generated 500 data sets with three different sample sizes (100, 500, 1000) and *p* = 20 variables. Six out of 20 variables were considered as the important variables (informative) and the rest of them were considered as the non-informative variables. Of 6 informative variables, four variables were considered as qualitative variables (one binary and three multinomial variables) generated from multinomial distribution and two were generated from the standard normal distribution. The regression coefficients for the informative variables varied between − 1 and 1 and they were considered as zero for the non-informative variables. The response variable was generated using logistic distribution. We used sensitivity and specificity to compare three different methods of variable selection. The sensitivity showed the proportion of informative variables correctly selected by the model and specificity showed the proportion of non-informative variables not selected by the model correctly (not selecting non-informative variables) [29].

### Software

Data entering and calculation of descriptive statistics were done using SPSS 24.0 and all other analyses were conducted using R 3.5.2 software by “grpreg” package (version 3.2–1) [30].

## Results

Table 1 shows the characteristics of the 1259 participants included in this study. For example, about 61% of the students were female. The average age of the students was 22.54 ± 3.34 (mean ± standard deviation) years. According to the cutoff point of 23 for a total score of psychiatric distress, 518 (41.14%) students had psychiatric distress.

In this study, the prediction performances of the three approaches of the stepwise logistic regression, penalized logistic regression with group LASSO penalty and penalized logistic regression with group SCAD penalty were compared using different criteria over the testing set. Table 2 shows the results. According to the results in Table 2, using group SCAD and the group LASSO penalties for the variable selection resulted in comparable prediction performances in terms of sensitivity, specificity, LR+ and LR- and total accuracy. However, the group SCAD penalty selected a fewer number of variables (11 out of 29) compared to the group LASSO (16 out of 29). Moreover, the stepwise approach provided a slightly better sensitivity (0.516), however, its specificity was very low (0.151).

Therefore, we continued to analyze the data using the group SCAD because it provided the same results with a fewer number of variables. Table 3 shows the associations of the selected variables by group SCAD and psychiatric distress among college students. According to the results shown in Table 3, having an average grade less than 14 in the previous semester (OR = 2.57; 95% CI: 1.18, 5.58), being a BSc student (OR = 0.32; 95% CI: 0.15, 0.65), being optimistic about the future (OR = 0.64; 95% CI: 0.44, 0.94), having a boy/girlfriend (OR = 1.63; 95% CI: 1.24, 2.14), having an emotional breakup (OR = 1.82; 95% CI: 1.37, 2.41), smoking an average daily number of cigarettes between 1 and 9 (OR = 1.58; 95% CI: 1.04, 2.41), substance abusing during previous month (OR = 2.55; 95% CI: 1.32, 4.93), and having suicidal thoughts ever (OR = 5.75; 95% CI: 3.84, 8.61) were correlated with psychiatric distress significantly.

**Table 3:** Correlates of psychiatric distress^a^ among college students selected by group SCAD analysis

Variable		Unadjusted			Adjusted	
	OR	OR (95% CI)	***P***-value	OR	OR (95% CI)	***P***-value
**Intercept**				1.21		0.704
**City**						
Towns of other provinces (Reference category)	1.00			1.00		
Home town	0.84	(0.64, 1.11)	0.227	0.87	(0.62, 1.20)	0.406
Surrounding town	1.20	(0.91, 1.57)	0.181	1.30	(0.95, 1.77)	0.102
**College**						
Rehabilitation (Reference category)	1.00			1.00		
Dentistry	1.16	(0.58, 2.31)	0.674	0.68	(0.24, 1.90)	0.470
Public health	1.26	(0.67, 2.35)	0.461	0.72	(0.35, 1.46)	0.370
Medicine	0.96	(0.52, 1.76)	0.899	0.58	(0.22, 1.56)	0.287
Nursing/Midwifery	1.12	(0.59, 2.15)	0.715	0.85	(0.41, 1.77)	0.682
Paramedical	1.29	(0.69, 2.39)	0.421	0.90	(0.44, 1.83)	0.781
Pharmacology	2.09	(1.02, 4.27)	0.042	1.69	(0.56, 5.03)	0.345
**The average grade of the previous semester**						
≥ 18 (Reference category)	1.00			1.00		
< 14	4.71	(2.43, 9.11)	< 0.001	2.57	(1.18, 5.58)	0.017
14–15.99	1.48	(1.02, 2.16)	0.039	1.22	(0.79, 1.90)	0.361
16–17.99	1.25	(0.88, 1.77)	0.205	1.27	(0.84, 1.90)	0.243
**Educational level**						
Ph.D. (Reference category)	1.00			1.00		
BSc	0.41	(0.22, 0.77)	0.006	0.32	(0.15, 0.65)	0.002
MSc	1.32	(0.63, 2.75)	0.456	0.78	(0.34, 1.80)	0.574
MD	0.44	(0.23, 0.83)	0.012	0.42	(0.16, 1.08)	0.074
**Interest in the discipline**						
No (Reference category)	1.00			1.00		
Yes	0.42	(0.31, 0.56)	< 0.001	0.79	(0.53, 1.19)	0.274
**Optimistic about the future**						
No (Reference category)	1.00			1.00		
Yes	0.44	(0.33, 0.58)	< 0.001	0.64	(0.44, 0.94)	0.025
**Having a boy/girlfriend**						
No (Reference category)	1.00			1.00		
Yes	2.26	(1.79, 2.85)	< 0.001	1.63	(1.24, 2.14)	< 0.001
**Having an emotional breakup**						
No (Reference category)	1.00			1.00		
Yes	2.73	(2.14, 3.47)	< 0.001	1.82	(1.37, 2.41)	< 0.001
**Number of cigarettes per day**						
Non-smoker (Reference category)	1.00			1.00		
1–9	3.07	(2.17, 4.33)	< 0.001	1.58	(1.04, 2.41)	0.031
≥ 10	3.42	(1.64, 7.14)	0.001	1.01	(0.39, 2.59)	0.974
**Substance abuse previous month**						
No (Reference category)	1.00			1.00		
Yes	6.96	(3.99, 12.14)	< 0.001	2.55	(1.32, 4.93)	0.005
**Having suicidal thoughts ever**						
No (Reference category)	1.00			1.00		
Yes	7.83	(5.43, 11.29)	< 0.001	5.75	(3.84, 8.61)	< 0.001

^a^Based on GHQ-28 questionnaire

Table 4 shows the results of the simulation study. According to the results, the sensitivities of the group LASSO and group SCAD were comparable for different sample sizes, however, the specificity of the group SCAD was much greater (0.831 vs. 0.433 for the sample size of 1000). For the small sample size (*n* = 100), both sensitivities and specificities were moderate. In all the three scenarios, the SCAD selected a smaller number of variables compared with the LASSO. As seen, the performance of the stepwise regression was poor in terms of identifying informative variables.

**Table 4:** Results for various methods in the simulation study for different sample sizes and 6 relevant variables (*p* = 20) over 500 replicates

	Variable selection method	NO. selected variables	Sensitivity	Specificity
	**SCAD**	4.00	0.646	0.890
		(2.895)	(0.241)	(0.096)
**n = 100**	**LASSO**	8.39 (4.735)	0.767 (0.254)	0.663 (0.208)
	**Stepwise**	7.30	0.183	0.609
		(2.134)	(0.112)	(0.119)
	**SCAD**	9.38	0.991	0.752
		(2.23)	(0.045)	(0.161)
***n*** **= 500**	**LASSO**	13.37	0.999	0.473
		(2.571)	(0.107)	(0.010)
	**Stepwise**	7.55	0.212	0.612
		(1.565)	(0.107)	(0.083)
	**SCAD**	8.56 (1.56)	1.00 (0.00)	0.831 (0.116)
***n*** **= 1000**	**LASSO**	14.93	1.000	0.433
		(2.54)	(0.00)	(0.182)
	**Stepwise**	5.05	0.238	0.639
		(1.411)	(0.093)	(0.077)

Values in parenthesis are standard deviations over 500 repetitions

## Discussion

In the present study, we utilized a penalized approach to select the correlates of psychiatric distress among college students called group SCAD. This approach was used to conduct variable selection and parameter estimation, simultaneously. We used a real dataset to investigate and to compare the performances of the group SCAD, the group LASSO, and the stepwise logistic regression methods. The findings of the present study showed that the group SCAD and the group LASSO outperformed the traditional stepwise approach in terms of prediction accuracy. Our results showed that the educational variables including the average grade and educational level, being optimistic about the future and high-risk behaviors including having a boy/girlfriend, having an emotional breakup, the average daily number of smoked cigarettes, substance abusing during previous month and having suicidal thoughts were significantly associated with psychiatric distress among college students.

The findings of the present study showed that there were positive relationships between the daily number of smoking and drug abusing and psychiatric distress, such that smoking and drug abusing increased the chance of having psychiatric distress by 1.58 (for those who smoked 1–9 cigarettes per day) and 2.55 times, respectively. These findings are consistent with those of previous studies, conducted on students (schools and high schools). A study conducted on 1515 students (aged 15–18) in Glasgow (the West of Scotland), by Green et al., indicated that students who smoked had increased levels of distress [13]. Another study conducted on 13,486 students (aged 6–18) in Iran, by Kelishadi et al., also indicated that smoking increased the risk of having angriness, worrying, anxiety, the victim and bully behaviors [31]. Smoking and substance abusing were also associated with psychological distress in the results of Poorolajal et al. [7]. Beside the student population [32, 33], the same findings have been found in other general populations as well [14, 16]. These consistencies indicate that there is a need to consider strategies that address mental health issues as well as smoking/drug abusing prevention programs as parts of college health and consulting services.

We also found that having suicidal ideation was positively associated with psychiatric distress among college students (it increased the chance of psychiatric distress by 5.75 times), which was in agreement with the results of other studies. A study, conducted by Eskin eta al on university students (including 12 countries of Austria, China, Iran, Italy, Japan, Jordan, Palestine, Saudi Arabia, Tunisia, Turkey, the UK, and the United States), indicated that psychological distress was significantly associated with suicidal thoughts and attempts [34]. Other studies reported the same findings [7, 35, 36]. Moreover, it has been reported that several high-risk behaviors like substance/alcohol abusing as well as smoking increased the risk of suicidal thoughts/attempts [37–39]. Moreover, other risky behaviors like having a boy/girlfriend and having an emotional breakup were positively associated with psychological distress (increased the odds of psychological distress by 1.63 and 1.82 times respectively). These findings were also consistent with the results of other studies [40]. The emotional breakup has been reported to be associated with a decrease in well-being level [41], lower satisfaction of life [42] and rage and sadness [43]. On the other hand, being optimistic about the future was associated with a lower chance of psychological distress among students. The results of a study, conducted on college students in the United States, showed that higher optimism and self-esteem were associated with lower levels of mental distress among college students [44]. Poorolajal et al. also reported that being optimistic about the future was negatively associated with psychological distress [7]. Furthermore, our findings showed that educational variables including grade point average and educational level were associated with psychological distresses, such that students with average grade points less than 14 were 2.57 times more likely to have psychological distress and Ph.D. students were 3.12 times more likely to have psychological distress. This finding was also in agreement with the results of other studies. Lipson et al. conducted a study on 43,210 students in the US and found that students in doctorate-granting institutions were at a higher risk of mental health problems [45]. Levecque et al. also showed that half of PhD students experience psychological distress and one-third of Ph.D. students are at risk of a common psychiatric disorder. Moreover, they showed that the prevalence of mental health problems is higher among Ph.D. students compared with the highly educated general population, and higher education students [46].

The performance of the statistical methods used in this study has been investigated by several studies in terms of selecting important variables. Ogutu and Piepho compared different penalized methods like group SCAD, group LASSO and the minimax concave penalty (MCP) and concluded that all the penalized methods produced satisfactory predictive accuracies for most practical purposes [47]. Morozova et al. conducted a simulation study and showed that model selection with stepwise methods is highly unstable compared with the penalized methods [48].

Our simulation study showed that the group SCAD penalty performed very well in terms of identifying informative variables and had smaller false positives compared with the group LASSO. Moreover, stepwise regression failed in identifying important variables. We considered only independent variables and it is suggested to consider correlated scenarios in the future studies. Hastie et al. compared the stepwise regression and LASSO penalized method and concluded that the LASSO method is preferred [49]. Lu et al. showed, through simulation studies, that the penalized methods of SCAD and LASSO are preferred to the stepwise regression model; as they have greater true positives and smaller false negatives [50].

There were some limitations to the present study. First, there were some sensitive questions in the used self-reported questionnaire about sexual activities. They lead to underestimations for those variables (estimation bias). Second, questions about alcohol use (and binge drinking) were missed in this study, which is likely associated with the outcome of interest and a public health concern among young people [51, 52] and it is suggested to be considered in the future studies. Third, in this study, it was not possible to obtain cause-effect relations between explanatory variables and the outcome as this was a cross-sectional study. So, whether the outcome of this study caused high-risk behaviors (or vice versa) is not evident. Another limitation was that the current study involved voluntary subjects (i.e., highly motivated), with the majority being female, and 41% of the sample reported the MD educational level. This may prone our estimations to the selection bias problem. Moreover, we used simple imputation in this study that can add some biases to the estimations. It is suggested to use multiple imputations using generalized linear models to reduce the effect of this bias. Despite these limitations, we used an appropriate statistical method to select variables that are correlated with the binary outcome variable. This allows us to select associated variables more reliably compared to the other traditional methods, like conducting the stepwise logistic regression or choosing included variables in a multiple logistic regression through a univariate screening procedure. Our used method also allows for considering all the two-way or higher-order interactions between the variables in the model and to set penalty terms on them without any limitation. The used approach (penalized logistic regression) can handle high dimensional settings, while the stepwise technique cannot deal with this situation and it may provide unstable results. According to theoretical studies, the group SCAD penalty enjoys oracle property which indicates that this method can select true influential variables consistently [24].

## Conclusions

The present study used a statistical method to investigate and to identify associated variables of mental health issues among college students in Iran. Overall, through real data analysis and simulation studies, it was shown that the penalized logistic regression method should be considered as plausible alternatives to the traditional stepwise regression. Several correlates for psychological distress, identified in this study, highlights the necessity of paying attention to the mental health requirements of young adults when entering college and our results can be used by policymakers.

## Data Availability

The data are available upon reasonable requests from the corresponding author.

## Abbreviations

*SCAD*: Smoothly clipped absolute deviation
*LASSO*: Least absolute shrinkage and selection operator
*LR+*: *Positive Likelihood Ratio*
*LR-*: *Negative Likelihood Ratio*
